# Digital phantom versus patient‐specific radiation dosimetry in adult routine thorax CT examinations

**DOI:** 10.1002/acm2.14389

**Published:** 2024-05-22

**Authors:** Antonios E. Papadakis, Vassiliki Giannakaki, John Stratakis, Marios Myronakis, Habib Zaidi, John Damilakis

**Affiliations:** ^1^ University Hospital of Heraklion Medical Physics Department Stavrakia, Heraklion Crete Greece; ^2^ Division of Nuclear Medicine and Molecular Imaging Geneva University Hospital Geneva Switzerland; ^3^ Department of Nuclear Medicine and Molecular Imaging University of Groningen University Medical Center Groningen Groningen Netherlands; ^4^ Department of Nuclear Medicine University of Southern Denmark Odense Denmark; ^5^ University Research and Innovation Center Obuda University Budapest Hungary; ^6^ University of Crete, Medical School Medical Physics Department Stavrakia, Heraklion Crete Greece

**Keywords:** CT, digital phantoms, dosimetry, experimental phantoms, Monte Carlo

## Abstract

**Purpose:**

The aim of this study was to compare the organ doses assessed through a digital phantom‐based and a patient specific‐based dosimetric tool in adult routine thorax computed tomography (CT) examinations with reference to physical dose measurements performed in anthropomorphic phantoms.

**Methods:**

Two Monte Carlo based dose calculation tools were used to assess organ doses in routine adult thorax CT examinations. These were a digital phantom‐based dosimetry tool (NCICT, National Cancer Institute, USA) and a patient‐specific individualized dosimetry tool (ImpactMC, CT Imaging GmbH, Germany). Digital phantoms and patients were classified in four groups according to their water equivalent diameter (*D*_w_). Normalized to volume computed tomography dose index (CTDI_vol_), organ dose was assessed for lungs, esophagus, heart, breast, active bone marrow, and skin. Organ doses were compared to measurements performed using thermoluminescent detectors (TLDs) in two physical anthropomorphic phantoms that simulate the average adult individual as a male (Alderson Research Labs, USA) and as a female (ATOM Phantoms, USA).

**Results:**

The average percent difference of NCICT to TLD and ImpactMC to TLD dose measurements across all organs in both sexes was 13% and 6%, respectively. The average ± 1 standard deviation in dose values across all organs with NCICT, ImpactMC, and TLDs was ± 0.06 (mGy/mGy), ± 0.19 (mGy/mGy), and ± 0.13 (mGy/mGy), respectively. Organ doses decreased with increasing *D*_w_ in both NCICT and ImpactMC.

**Conclusion:**

Organ doses estimated with ImpactMC were in closer agreement to TLDs compared to NCICT. This may be attributed to the inherent property of ImpactMC methodology to generate phantoms that resemble the realistic anatomy of the examined patient as opposed to NCICT methodology that incorporates an anatomical discrepancy between phantoms and patients.

## 1 INTRODUCTION

Monte Carlo (MC)‐based computational methods have been widely employed to estimate patient radiation dose in computed tomography (CT). These methods utilize an accurate in silico replication of the CT scanner geometry and digital phantoms. Libraries of digital phantoms representing the 10th, 50th, and 90th percentiles of standing height and body weight in Caucasian population have been constructed using scaling algorithms to match the anthropometric parameters of reference patients at various body sizes.^1^, ^2^, ^3^, ^4^ Although these phantoms are derived from a large amount of patient data and can simulate the average patient at a given body size, they cannot accurately represent the somatometrical characteristics and the actual anatomy of a real patient since small or bigger differences may always exist. Besides, patients referred to CT are commonly suffering from a pathology that may alter the shape, location, or composition of specific radiosensitive organs.

Several studies have documented that accurate patient‐specific, individualized dosimetry should be ideally performed on anatomic models produced directly from the tomographic images of the exposed patient.^5^, ^6^, ^7^, ^8^ Such models can more closely replicate the unique silhouette and can realistically mimic the anatomy of the examined individual. Besides, these anatomic models can accurately represent the shape, size, and location of each radiosensitive organ in the human body including any anatomical abnormalities related to tissue pathology of the examined patient. While the digital phantoms have been widely used to estimate radiation dose in CT, to the best of our knowledge, a comparison between digital phantom based and patient‐specific, anatomic model based, individualized dosimetry has not yet been reported.

The aim of this study was to compare the organ doses estimated through digital phantom based and patient‐specific based dosimetric methods using as a reference physical dose measurements performed in two anthropomorphic phantoms representing a male and a female adult individual with the average somatometrical characteristics.

## 2 METHODS

This is a retrospective study that was approved by our institutional review board. This study is compliant with the Declaration of Helsinki on ethical principles for medical research involving human subjects. Informed consent was waived.

### 2.1 Digital phantom‐based dosimetry

**FIGURE 1:**
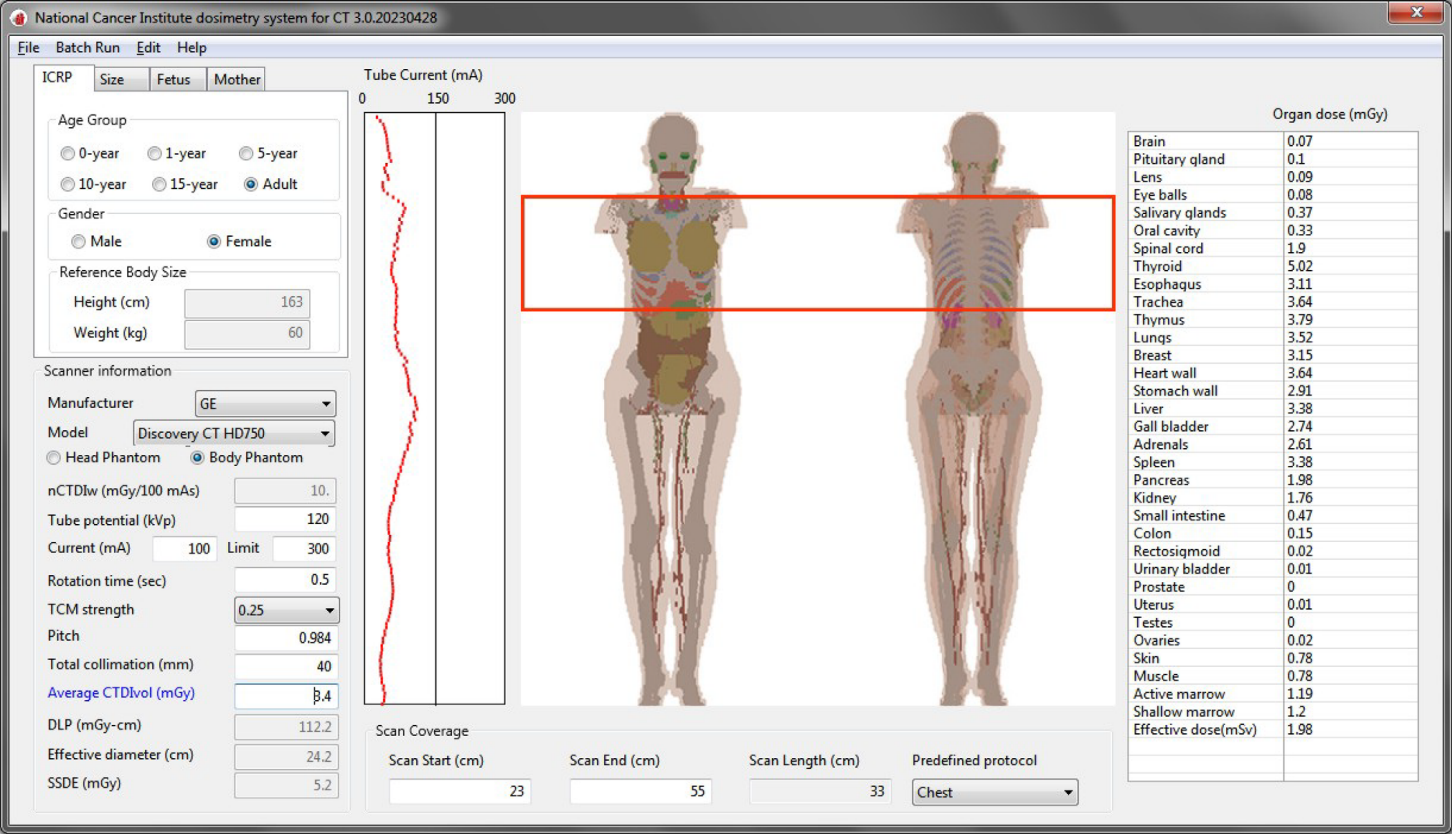
The graphical user interface of the NCICT 3.0 dose calculator tool where height and weight of the computational model and CT exposure parameters are input to derive organ doses in routine thorax CT imaging.

The CT dose calculator tool from National Cancer Institute dosimetry system was used (NCICT, version 3.0.20230428, National Cancer Institute, USA). This tool provides absorbed doses to radiosensitive organs and tissues based on a pre‐calculated dose database and a library of reference voxel models that represent patients at various heights and weights at both sexes (Figure 1).^9^, ^10^, ^11^ The dosimetric results contained in the database have been validated through experimental measurements conducted using physical anthropomorphic phantoms combined with dosimeter detectors.^12^, ^13^, ^14^, ^15^ This tool constitutes a standalone software that runs on a personal computer and is provided at no charge by the National Cancer Institute, when used for academic non‐commercial research purposes.

Normalized to CTDI_vol_ dose values ($nOD_{T,NCICT}$) for each organ (T) were retrieved from the NCICT pre‐calculated dose database for adult thorax CT performed on a Discovery 750HD scanner from General Electric Healthcare (Milwaukee, USA). The exposure parameters were as follows: 120 kVp, beam collimation 40 mm, large bowtie filter, and pitch 0.984. Organ dose was retrieved from acquisitions performed with i) fixed tube current (mA) set to deliver CTDI_vol_ 12 mGy and the “strength” parameter set at 0, and ii) tube current modulation (TCM) with the “strength” parameter set at 0.25. This value was selected because it reduced the originally selected CTDI_vol_ by 24%, which is the dose reduction that has been commonly achieved in thorax TCM acquisitions.^16^ It should be noted that the “strength” parameter used by NCICT is not relevant to modulation strength setting employed in TCM acquisitions by specific CT vendors. The tube current limit, which was an additional parameter required by the NCICT tool, was set at 500 mA. The scanning length for thorax was set automatically on the body habitus of each model. $nOD_{T,NCICT}$ was retrieved for lungs, esophagus, heart, breast, active bone marrow, and skin for the entire skeleton in each one of the 195 adult reference voxel phantoms included in the library. There were 101 male and 94 female digital phantoms. The mean height and weight were 1.74 m ± 0.1 m (range: 1.60–1.90 m) and 83.1 kg ± 21.9 kg (range: 50–125 kg) for males. The corresponding values for females were 1.62 m ± 0.09 m (range: 1.50 –1.75 m) and 76.8 kg ± 24.0 kg (range: 40–120 kg). The body mass index (BMI, kg/m^2^) of each phantom was calculated as the ratio of the weight to the square of height. To estimate water equivalent diameter (*D*_w_, cm) of thorax at each digital phantom, the following equation was used:

(1) $$D_{w}=0.447\times BMI+16.7$$

This equation has been the result of a study performed to investigate the correlation between BMI and *D*_w_ of various anatomical regions in 193 adult patients at various body sizes.^17^

### 2.2 Patient‐specific individualized dosimetry

**FIGURE 2:**
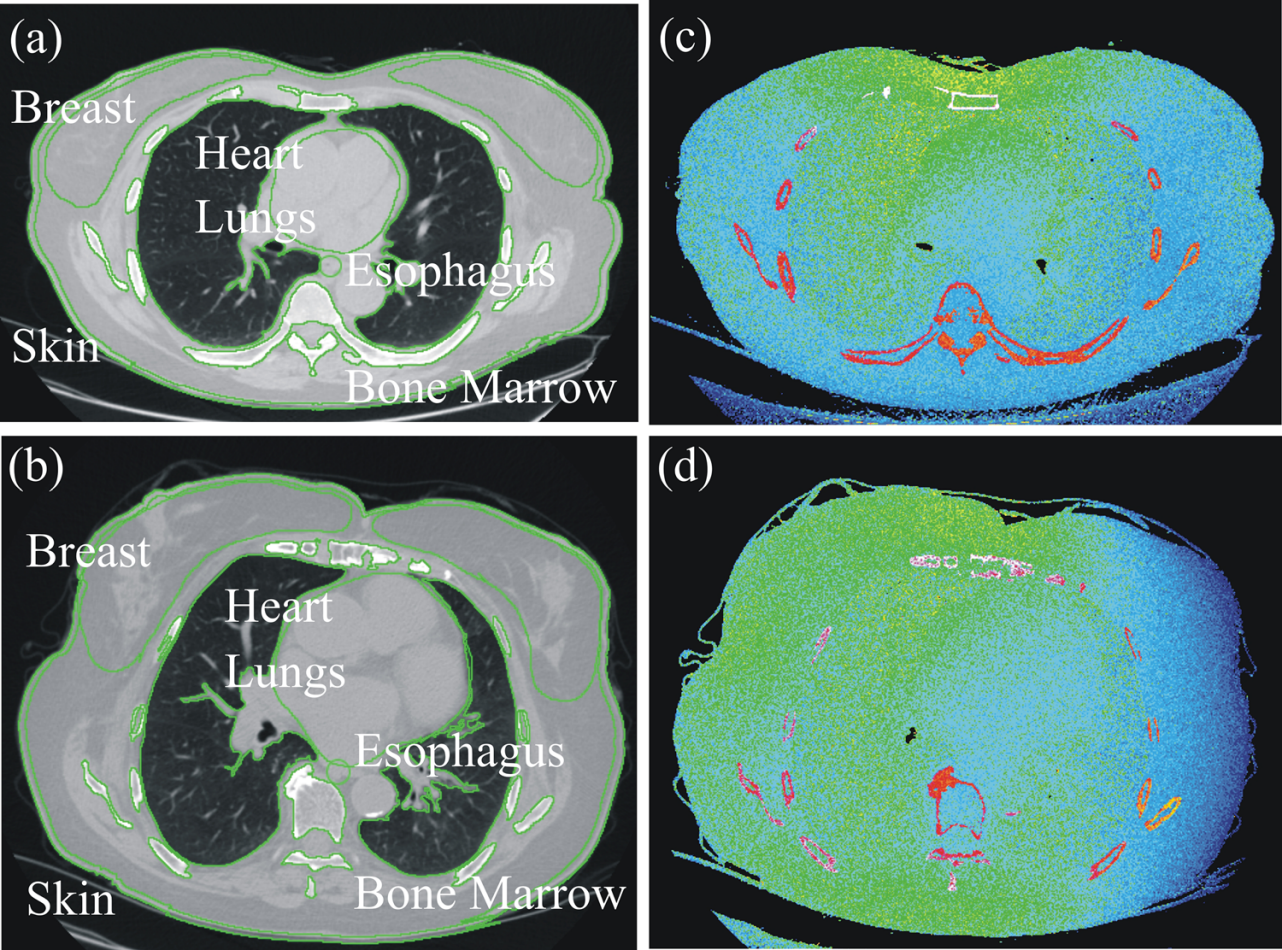
The axial slice within the CT image series used to create patient‐specific digital phantom of a 35‐year‐old (a), and a 48‐year‐old (b) female along with the corresponding color coded output dose images generated through patient‐specific dosimetry (c) and (d). The calculated *D*_w_ was 27.1 cm both for (a) and (b).

A MC‐based simulation software package that enables the computation of the radiation dose imparted in any patient referred to CT (ImpactMC, CT Imaging GmbH, Erlangen, Germany) was used. This package has been validated and employed in several dosimetric research studies.^18^, ^19^, ^20^ The software uses the CT images of the examined patient as input to create a patient‐specific anatomic model (Figure 2a,b). Image series from 92 consecutive routine thorax CT examinations were used to produce 92 patient‐specific phantoms at a voxel resolution of 0.97 × 0.97 × 2.5 mm. There were 59 males and 33 female patients. The CT scanner modeled was a Revolution HD (General Electric Healthcare, Milwaukee, USA). The scanning parameters were those prescribed by the manufacturer's routine examination protocols: 120 kVp, beam collimation 40 mm, large bowtie filter, reconstruction slice width 2.5 mm, and pitch 0.984. Acquisitions were simulated from lung apices to the 12th rib. Simulated scanning length included the imaged volume without accounting for the z‐overscan distance. Simulations were performed for each patient‐specific phantom placed at the scanner's isocenter with (i) fixed mA at CTDI_vol_ 12 mGy, and (ii) TCM. The mA(z) profile derived from the mean mA values listed in the patient images’ DICOM header was used. mA(z) profile values were retrieved from the DICOM header of the patient image data using an ImageJ plugin (version 1.46r, NIH, USA). The number of photons per simulation was 10^10^ and the simulation error was < 1 %. Following the MC simulation of each patient's phantom, an output image series was generated. These images map the normalized to CTDI free‐in‐air (CTDI_F_; mGy/mGy∙100 mAs) dose distribution (ND_F_) imparted in the patient's body, in voxel‐to‐voxel correspondence to the input CT image series (Figure 2c,d). Calculated absorbed dose ND_F_(T) for each organ (T) was normalized to the scanner reported CTDI_vol_ (mGy/100 mAs) for the 32 cm diameter phantom: $nOD_{T,ImpactMC}=ND_{F}\left(T\right)/CTDI_{vol}$. $nOD_{T,ImpactMC}$ was assessed for lungs, esophagus, heart, breast, active bone marrow, and skin in the primary irradiated skeletal sites. $nOD_{T,ImpactMC}$ was determined as follows: a ROI delineating the organ of interest was drawn on the original CT image (Figure 2a,b). For lungs and active bone marrow, ROI delineation was performed using thresholding‐based segmentation. The skin surface was contoured using thresholding‐based segmentation in each image slice. Skin ROI was then determined as the area outlining a strip at 3 mm distance inwards from skin surface throughout the circumference of the body cross‐section. ROI per CT image was pasted to the corresponding output dose‐image. The mean pixel value of the latter ROI was the mean normalized dose for the fraction of the organ depicted in that particular image. $OD_{T,ImpactMC}$ was estimated as the sum of the area‐weighted mean ROI values derived from all dose‐images where the organ of interest was depicted.

*D*_w_ was calculated for each patient according to the following equation:

(2) $$D_{w}=2\sqrt{\left[\frac{HU_{ROI}}{1000}+1\right]\times \frac{A_{ROI}}{\pi }}$$

where HU_ROI_ is the mean CT number measured within a ROI containing all image pixels depicting the patient's cross‐section, and *A*_ROI_ is the area of the body cross‐section contained within the ROI.^21^ *D*_w_ was measured at the level of the central axial plane depicting the heart.

### 2.3 Classification of digital phantoms and patients based on *D*_w_

To enable a comparison of organ doses between digital phantom‐based dosimetry and patient‐specific individualized dosimetry, male and female reference voxel digital phantoms and patients were classified in four groups based on their *D*_w_ as follows: group 1: *D*_w_ ≤ 26 cm, group 2: 26 cm < *D*_w_ ≤ 28 cm, group 3: 28 cm < *D*_w_ ≤ 31 cm, and group 4: 31 cm < *D*_w_ ≤ 35 cm for males, and *D*_w_ ≤ 24 cm, group 2: 24 cm < *D*_w_ ≤ 26 cm, group 3: 26 cm < *D*_w_ ≤ 28 cm and group 4: 28 cm < *D*_w_ ≤ 31 cm for females. For each group, average organ dose ± standard deviation (SD) values were estimated through digital phantom‐based dosimetry and patient‐specific individualized dosimetry. The coefficient of variation (CV) in each organ dose value was calculated as the ratio of the SD to the average organ estimated within each *D*_w_ group.

### 2.4 Thermoluminescent detector dosimetry and individualized dosimetry on physical anthropomorphic phantoms

**FIGURE 3:**
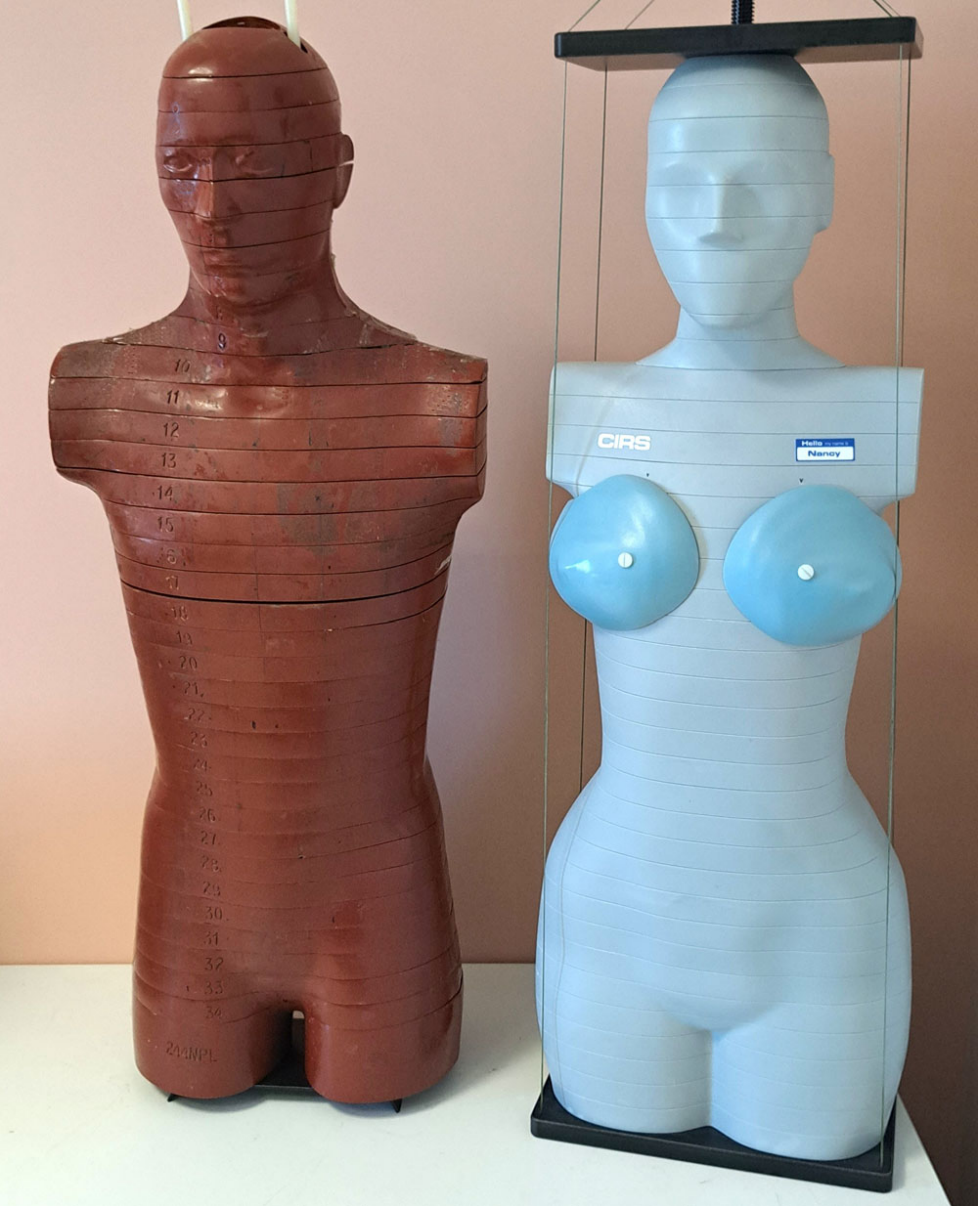
The male (left) and female (right) anthropomorphic phantoms used for physical absorbed dose measurements using thermoluminescent detectors.

Two physical anthropomorphic phantoms that simulate the average adult individual as a male, 1.73 m in height and 73.5 kg in weight (Alderson Research Labs, Stanford, California, USA) and as a female 1.60 m in height and 55 kg in weight (ATOM Phantoms, model 702‐D, CIRS Norfolk, USA), were used (Figure 3). The male phantom is composed of bone with a natural skeleton, soft tissue, and lung tissue equivalent materials. The female phantom is composed of artificial skeleton and equivalent materials for the average soft tissue, average bone tissue, cartilage, spinal cord, spinal disks, lung, brain, and sinus tissue. To create bone tissue, averaged mineral density of cortical and trabecular bone ratios are used. Active bone marrow tissue‐equivalent material is included in cranium, scapulas, clavicle, sternum, ribs, spine, sacrum, pelvis, and femura. Supine breasts, 1200 cm^3^ in volume, manufactured by 50% glandular and 50% adipose equivalent material, were attached on the female phantom to represent the clinically relevant geometry of a patient laying on her back (ATOM Phantoms, CIRS Norfolk, US7A). To classify each anthropomorphic phantom according to *D*_w_ grouping described in the previous section, *D*_w_ was calculated following the Equation (2) at the level of the middle axial plane outlining the heart. The phantoms are sectioned into multiple 2.5 cm thick, slabs. Each slab is drilled in holes, 5 mm diameter, to allow the placement of thermoluminescent detectors (TLDs). Holes are distributed across each slab in accordance with the location of specific radiosensitive organs. Sixty‐five TLD chips (TLD‐100H, Harshaw) were used to measure the absorbed dose to lungs, esophagus, heart, breast, and active bone marrow in the primary irradiated skeletal sites.

Skin dose was determined from longitudinal arrays of eight TLDs placed along the anterior, posterior, and lateral surfaces of each phantom. Measurements were performed based on the methods described in previous reports.^22^, ^23^, ^24^ The phantoms were subjected to a routine thorax CT acquisition on a Revolution HD, General Electric CT scanner. To facilitate comparison of the measured doses with corresponding doses assessed with the computational methods, acquisitions were performed with (i) fixed mA at CTDI_vol_ 12 mGy and (ii) TCM. To increase the measured signal and limit the coefficient of variation in the response among TLD chips, each CT scan was repeated 10 times. To determine the dose measured by each chip, the background signal was subtracted from the reading value and the latter was divided by the number of scans.

Before the exposure, the dosimeters were annealed for 10 min at 400°C and then slowly cooled to room temperature. A Harshaw‐3500 reader (Thermo‐Fisher Scientific, Waltham, Massachusetts, USA) was used to readout the irradiated dosimeters. Dose calibration of TLD chips was performed on the employed CT scanner free‐in‐air using a recently calibrated pencil‐shaped ionization chamber (DCT 10 RS Lemo, RTI Electronics AB, Sweden). The calibration coefficient (CC) of each TLD was determined as the ratio of the dose to air measured with the ionization chamber divided by the TLD signal. On the basis of their sensitivity, TLDs were grouped into batches so that the standard deviation of each batch was less than 3%.

Conversion of the TLD signal (TLD_Signal_) to absorbed dose (*D*_x_) was performed using the following equation:

(3) $$D_{x}=CC\times \frac{{\left(\mu_{en}/\rho \right)}_{T}}{{\left(\mu_{en}/\rho \right)}_{air}}\times TLD_{Signal}$$

where ${\left(\mu_{en}/\rho \right)}_{T}$ is the mass energy absorption coefficient of the organ/tissue at interest and ${\left(\mu_{en}/\rho \right)}_{air}$ is the mass energy absorption coefficient of air. Mass energy absorption coefficient values for soft tissue, lung, and air were derived from the National Institute of Standards and Technology (NIST) library.^25^ Normalized to CTDI_vol_ organ absorbed dose ($nOD_{T,TLDs}$) was calculated using the following equation:

(4) $$nOD_{T,TLDs}=\sum_{i=1}^{k}f_{i}\times \left[\frac{1}{\lambda }\sum_{j=1}^{\lambda }D_{x}^{j}\right]$$

where k is the total number of phantom slices covered by the organ at interest, *f*_i_ is volume fraction of each organ that was included in a single phantom slice, λ is the total number of TLD chips per phantom slice located within the organ at interest, and $D_{x}^{j}$ is the dose measured by the j_th_ TLD chip. Dose to active bone marrow was calculated using the TLD_Signal_ recorded from chips located in sternum, scapulas, clavicle, ribs, and thoracic spine. To take into account for the small dose enhancement originating from the secondary photoelectrons emitted from more highly attenuating trabecular bone that impart dose in the adjacent marrow tissue, the percentage excess dose factor of 15% reported by King et al.^26^ was used. The uncertainty of the measured organ dose values $SD(OD_{T,TLDs})$ was estimated according to the following equation:

(5) $$\begin{array}{ccc} & & SD\left(nOD_{T,TLDs}\right)\\ & & =\sqrt{{\left(SD(CC)\right)}^{2}+{\left(SD(TLD_{Signal})\right)}^{2}} \end{array}$$

where SD(CC) is the uncertainty in the calibration coefficient CC, which is 3%, and $SD(TLD_{Signal})$ is the uncertainty of the TLD measurement. A total of 30 TLDs belonging to the same batch were irradiated to the same amount of dose using the conventional x‐ray tube and then were readout. This procedure was repeated 10 times. The $SD(TLD_{Signal})$ was taken as the SD of the measured mean TLD_Signal_.

The patient‐specific individualized dosimetric methodology described in Section 2.2 was repeated using the axial CT image series derived from the male and female physical anthropomorphic phantoms. The anatomic models of the anthropomorphic phantoms were created using the MC based simulation software package (ImpactMC, CT Imaging GmbH, Erlangen, Germany). Image series that map the normalized to CTDI free‐in‐air dose distribution in voxel‐to‐voxel correspondence to the input CT image series of the anthropomorphic phantoms were generated. The size and position of each organ were determined in accordance to manufacturer specifications and previously published data.^27^ $nOD_{T,ImpactMC}$ values were determined for lungs, esophagus, heart, breast, active bone marrow and skin.

### 2.5 Comparison of organ doses determined through different dosimetric methodologies

Organ dose values derived from TLD measurements were compared to corresponding values calculated through patient‐specific individualized dosimetry on the anthropomorphic phantoms and values retrieved from digital phantoms that most closely matched the height and weight characteristics of the anthropomorphic phantoms. These were four digital phantoms for males: (i) 170 cm and 70 kg, (ii) i) 170 cm and 75 kg, (iii) 175 cm and 70 kg, and (iv) 175 cm and 75 kg, and one digital phantom for females; 160 cm, 55 kg. Moreover, organ dose values derived from TLD measurements on anthropomorphic phantoms were compared to corresponding average values calculated for the group of patients and retrieved for the group of digital phantoms that matched the corresponding *D*_w_ of the physical anthropomorphic phantoms.

## 3 RESULTS

Table 1 lists the $nOD_{T,NCICT}$ and $nOD_{T,ImpactMC}$ values for males and females classified in group 1. Table 1 also includes $nOD_{T,TLDs}$ values derived from physical measurements on anthropomorphic phantoms, both of which were classified in group 1 based on the calculated D_w_. The average ± 1 SD in dose values across all organs with NCICT, ImpactMC, and TLDs was ± 0.06 (mGy/mGy), ± 0.19 (mGy/mGy), and ± 0.13 (mGy/mGy), respectively.

**TABLE 1:** Normalized to CTDI_vol_ (mGy/mGy ± 1 SD) organ dose assessed through NCICT ($nOD_{T,NCICT}$), ImpactMC ($nOD_{T,ImpactMC}$), and physical measurements ($nOD_{T,TLDs}$) for males and females classified in group 1.

	NCICT		ImpactMC		TLDs^a^	
	Fixed mA	TCM	Fixed mA	TCM	Fixed mA	TCM
	Males					
Lungs	1.49 ± 0.06	1.51 ± 0.07	1.30 ± 0.34	1.12 ± 0.33	1.31 ± 0.13	1.11 ± 0.10
Esophagus	1.20 ± 0.07	1.22 ± 0.06	1.28 ± 0.15	1.11 ± 0.13	1.26 ± 0.12	1.15 ± 0.13
Heart	1.54 ± 0.09	1.51 ± 0.10	1.66 ± 0.17	1.25 ± 0.17	1.63 ± 0.15	1.20 ± 0.15
Active bone marrow	0.44 ± 0.02	0.45 ± 0.02	1.54 ± 0.20	1.40 ± 0.21	1.40 ± 0.13	1.11 ± 0.10
Skin	0.31 ± 0.02	0.32 ± 0.02	1.49 ± 0.20	1.29 ± 0.22	1.42 ± 0.14	1.24 ± 0.12
	Females					
Lungs	1.52 ± 0.08	1.59 ± 0.07	1.45 ± 0.21	1.38 ± 0.21	1.32 ± 0.13	1.15 ± 0.10
Esophagus	1.19 ± 0.08	1.25 ± 0.06	1.35 ± 0.13	1.25 ± 0.13	1.33 ± 0.13	1.23 ± 0.10
Heart	1.55 ± 0.08	1.59 ± 0.09	1.84 ± 0.18	1.55 ± 0.21	1.75 ± 0.15	1.37 ± 0.20
Breast	1.30 ± 0.10	1.32 ± 0.10	1.52 ± 0.17	1.38 ± 0.18	1.42 ± 0.13	1.25 ± 0.15
Active bone marrow	0.50 ± 0.04	0.52 ± 0.03	1.61 ± 0.15	1.51 ± 0.14	1.42 ± 0.12	1.51 ± 0.13
Skin	0.31 ± 0.02	0.32 ± 0.02	1.58 ± 0.21	1.34 ± 0.23	1.44 ± 0.14	1.25 ± 0.12

^a^ Calculated *D*_w_ for male and female anthropomorphic phantoms was 23.4  and 23.1 cm, respectively.

**FIGURE 4:**
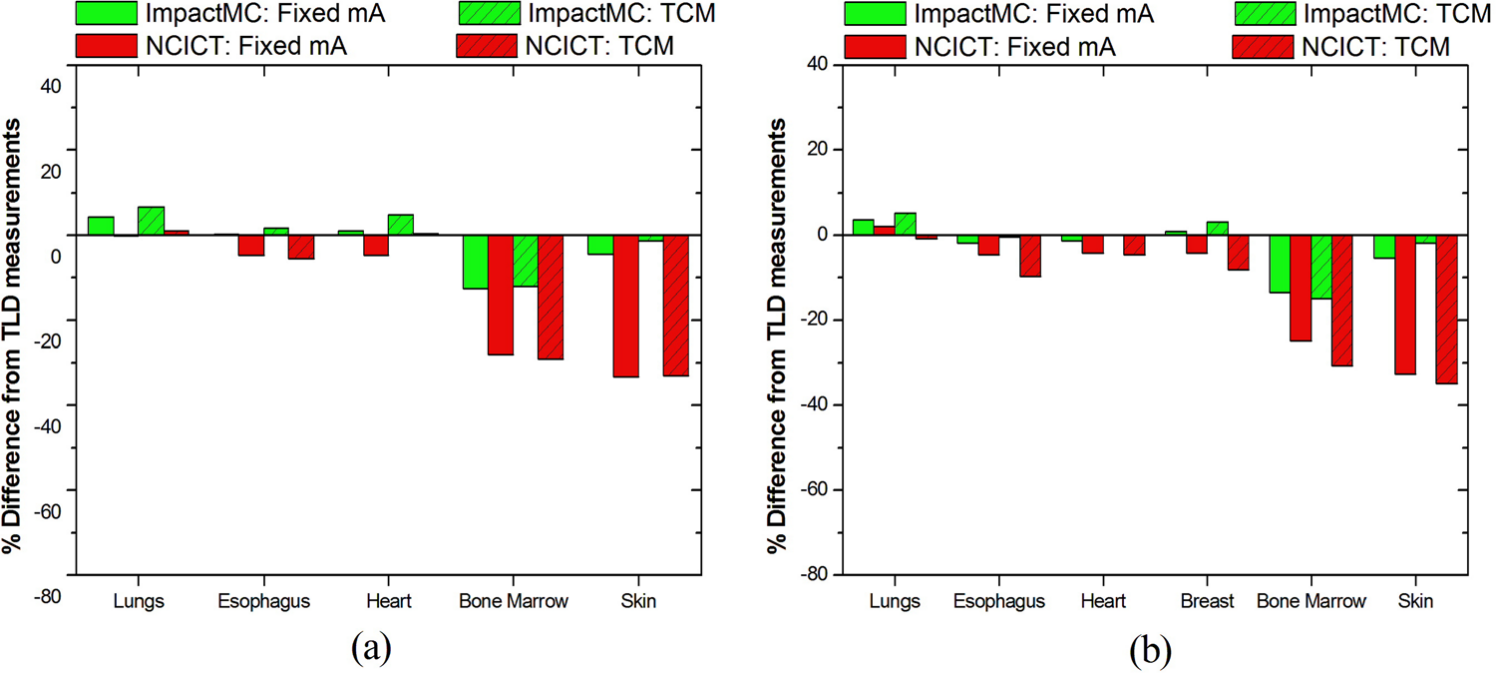
Percent differences in organ doses between different dosimetric methodologies employed using (i) NCICT along with the digital phantoms that matched the height and weight of anthropomorphic phantoms, (ii) ImpactMC along with the anatomic models of the male and female physical anthropomorphic phantoms and TLDs along with the male and female physical anthropomorphic phantoms. For each organ, the percent difference was estimated as: $\left(nOD_{T,Computed}-nOD_{T,TLDs}\right)\times 100/nOD_{T,TLDs}$, where $nOD_{T,Computed}$ is the normalized to CTDI_vol_ organ dose for organ T estimated with either NCICT or ImpactMC.

Figure 4a,b shows the percent difference between the organ dose values calculated using the anatomic models of the anthropomorphic phantoms (ImpactMC) and retrieved using the digital phantoms (NCICT) that matched the height and weight of the anthropomorphic phantoms, compared to corresponding doses measured with TLDs for males (a) and females (b). A close agreement was found between computed and measured values. The average percent difference of ImpactMC to TLD measurements across all organs in both sexes was 6%, whereas the corresponding difference for NCICT to TLD measurements was 13%. Active bone marrow and skin showed the highest difference among methods, with ImpactMC and NCICT having a difference from TLD measurements of 5% and 28% for active bone marrow and 4% and 33% for skin, respectively.

**FIGURE 5:**
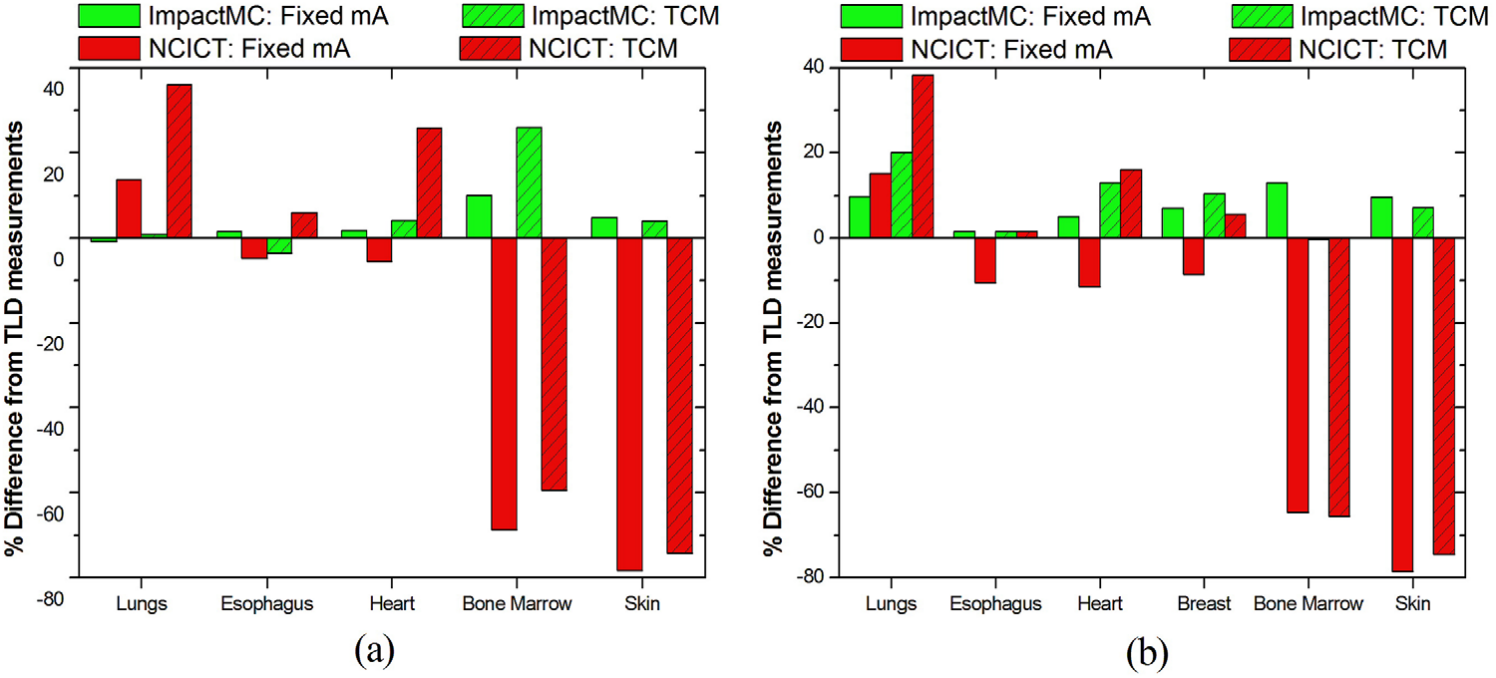
Percent differences in organ doses between ImpactMC and TLDs, and between NCICT and TLDs for male (a) and female (b) patients of group 1. For each organ, the percent difference was estimated as: $\left(nOD_{T,Computed}-nOD_{T,TLDs}\right)\times 100/nOD_{T,TLDs}$, where $nOD_{T,Computed}$ is the normalized to CTDI_vol_ organ dose for organ T estimated with either NCICT or ImpactMC.

Figure 5a,b shows the percent difference in organ values determined by each method compared to doses measured with TLDs for male (a) and female (b) patients in group 1. The average percent difference of ImpactMC to TLD measurements across all organs in both sexes was 8% and corresponding difference for NCICT to TLD measurements was 38%. Active bone marrow and skin showed the highest difference among methods, with ImpactMC and NCICT having a difference from TLD measurements of 12% and 65% for active bone marrow and 4% and 77% for skin, respectively.

**FIGURE 6:**
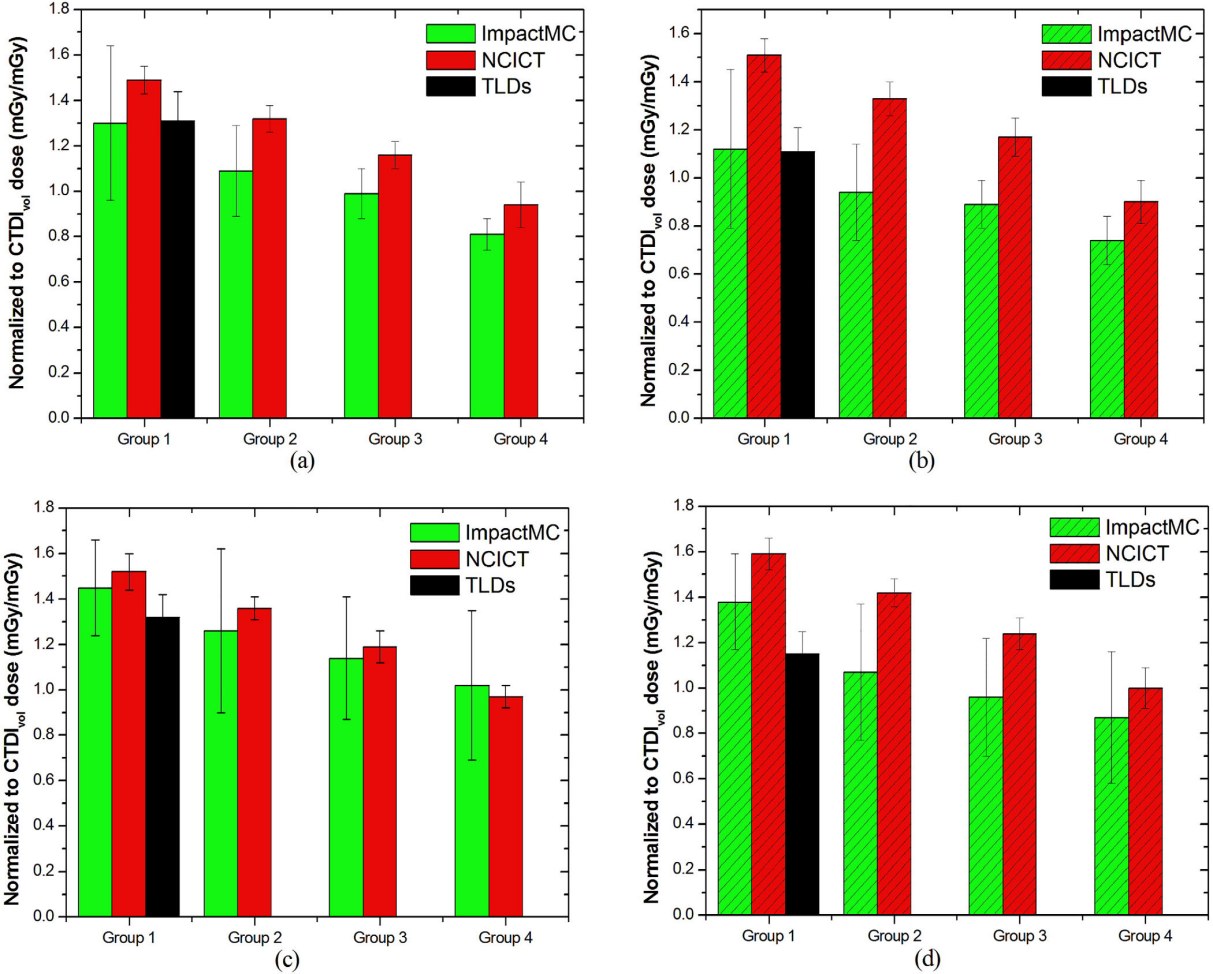
Normalized to CTDI_vol_ lung dose assessed through different methodologies for each group in males with fixed mA (a) and TCM (b) and females with fixed mA (c) and TCM (d).

Figure 6 shows the normalized to CTDI_vol_ lungs dose in fixed mA and TCM acquisitions for groups 1 through 4 of males and females. Lungs’ dose decreased with D_w_ in both NCICT and ImpactMC. Average CV values for lungs dose across all groups in males and females and in both acquisition modes were 6% for NCICT and 21% for ImpactMC. Results on normalized to CTDI_vol_ doses for esophagus, heart, breast, active bone marrow, and skin are shown in Figures S1–S5, respectively, of the supplemental material.

## 4 DISCUSSION

To the best of our knowledge, this is the first study, which compares organ doses estimated through a digital phantom‐based dosimetric tool and a patient specific‐based dosimetric tool. This comparison was done with reference to physical dose measurements performed in two anthropomorphic phantoms. Overall, there was a good level of agreement between doses measured and calculated using the software dosimetric tools. However, our results have shown that organ doses estimated with ImpactMC were in closer agreement to TLDs compared to NCICT. The average percent difference between TLDs and NCICT was, however, reduced when comparison was performed using the digital phantoms that matched the height and weight of the physical anthropomorphic phantoms. In fixed mA acquisitions, most organ doses estimated with ImpactMC for patients in group 1 were slightly higher than TLD measurements with an average variation of 4% in males and 7% in females. On the contrary, most organ doses estimated with NCICT for digital phantoms in group 1 were markedly lower than TLD measurements with an average variation of 23% in males and 24% in females.

Differences in estimated $nOD_{T,ImpactMC}$ and $nOD_{T,NCICT}$ with reference to $nOD_{T,TLDs}$, may be partly attributed to differences in the stature of the phantoms/models employed by each dosimetric method. Besides, organs and tissues in the anthropomorphic phantoms are made by homogenous density materials. In reality, however, tissues of non‐homogenous density can potentially alter the shielding on the surrounding organs, which may slightly affect the measured organ doses. The homogenous density material employed in the anthropomorphic phantoms also implies that hollow tissues, such as esophagus, are considered as solid organs.

Among examined organs, active bone marrow and skin showed the greatest average variation between NCICT and TLD measurements, whereas ImpactMC assessed dose, aligned well with TLD measurements. NCICT assesses active bone marrow dose for bone sites located in the entire skeleton, including the cranium, sternum, spine, ribs, pelvis, and femur, which undergo only secondary irradiation with scatter at a markedly reduced dose. Moreover, NCICT assesses skin dose for the skin area distributed throughout the entire body surface. On the contrary, both our calculations with ImpactMC and physical measurements with TLDs, considered only bone located in thoracic skeletal sites and skin in thorax anatomy, where bone marrow and skin are primarily irradiated. This practice was followed because patient‐specific MC organ dose estimation is limited to the scanning length and does not allow dose estimation for organs located outside the imaged volume. To facilitate a direct comparison of organ doses derived through ImpactMC and TLD methodologies, TLDs were placed in skeletal sites of the anthropomorphic phantoms that are primarily irradiated in thorax CT. Apparently, the discrepancy between NCICT and TLD measurements would be substantially reduced if TLDs were placed in sites throughout the whole skeleton of the anthropomorphic phantoms. Of note is also that the bone marrow and skin dose values determined in this study with ImpactMC and TLDs should not be used to calculate effective dose, since this quantity requires dose values derived from organs distributed throughout the entire human body. The active bone marrow and skin dose values retrieved from NCICT would be more appropriate to calculate effective dose. However, for the purpose of radiogenic risk assessment using the BEIR VII coefficients for each primarily exposed radiosensitive organ, the organ dose values estimated through ImpactMC should be used.^28^

Of note is that ± SD values of $nOD_{T,ImpactMC}$ across all organs are higher than corresponding ± SD values of $nOD_{T,NCICT}$. For instance, $nOD_{T,ImpactMC}$ and $nOD_{T,NCICT}$ for lungs in males of group 1 at fixed mA are 1.3 ± 0.4 (mGy/mGy) and 1.5 ± 0.06 (mGy/mGy), respectively. The increased ± SD in $nOD_{T,ImpactMC}$ values can be explained by the inherent ability of ImpactMC to create anatomic models that accurately match the realistic anatomical features of each patient's physical characteristics. Different patients may exhibit differences in the size, shape, or location of specific radiosensitive organs. Furthermore, patients referred to CT commonly suffer a pathology that may substantially alter the anatomy or composition of tissues. Figure 2 demonstrates two female patients at equal *D*_w_, who received substantially different organ doses due to differences in their anatomical stature. Specifically, estimated $nOD_{T,ImpactMC}$ for lungs and breast were 0.85 ± 0.19 and 1.08 ± 0.22 mGy/mGy, respectively, for female in Figure 2a versus 1.13 ± 0.18 and 1.26 ± 0.21 mGy/mGy, respectively, for female in Figure 2b. The digital phantoms employed by NCICT, on the contrary, utilize a series of phantoms that describe the human anatomy by combining mathematical equations along with non‐uniform rational basis‐spline and polygon mesh surfaces.^10^, ^11^, ^29^, ^30^ To model patients at various body sizes and take into account changes regarding the size of each organ, the phantoms are uniformly scaled. This process is limited in that it does not allow for changes in relative organ position, shape, or body anatomy that may be present in real patients, who commonly suffer changes in tissue characteristics owing to the presence of a pathology.

NCICT provided dose estimates that did not substantially vary between fixed mA and TCM acquisitions (Table 1). Typically, the $nOD_{T,NCICT}$ for male's heart was 1.54 ± 0.09 (mGy/mGy) and 1.51 ± 0.10 (mGy/mGy) in fixed mA and TCM, respectively. This finding is most likely due to the theoretical TCM model employed by the NCICT.^10^ This model is based on a generic modulation scheme that cannot exactly emulate the TCM operation of the CT scanner. As a result, this theoretical TCM model may not realistically adapt the mA to the anatomy of each phantom within the library of the digital phantoms. On the contrary, organ dose values estimated through ImpactMC and measured with TLDs showed a dose decrease in TCM compared to fixed mA acquisitions. It should be noticed that ImpactMC uses the realistic mA(z) profile derived from the DICOM header of each patient's CT image series. Besides, TLD dose measurements record the doses absorbed by each organ during TCM activated CT scans reflecting the behavior of the vendor's‐specific TCM algorithm in the clinical scenario.

Organ doses estimated by ImpactMC and NCICT indicated that $nOD_{T,ImpactMC}$ and $nOD_{T,NCICT}$ are reduced with increasing D_w_. This was not unexpected since organ dose per CTDI_vol_ is known to follow an exponential decay with increased body attenuation.^31^, ^32^, ^33^ Typically, $nOD_{T,NCICT}$ and $nOD_{T,ImpactMC}$ for breast in TCM acquisitions ranged from 1.32 ± 0.10 (mGy/mGy) to 0.97 ± 0.09 (mGy/mGy) and 1.38 ± 0.18 (mGy/mGy) to 0.87 ± 0.16 (mGy/mGy) for group 1 and group 4, respectively. Papadakis et al.^32^ have documented that correlation of this exponential decay is improved in TCM compared to fixed mA acquisitions. Khatonabadi et al.^34^ have demonstrated that normalized to CTDI_vol_ organ dose coefficients that account for TCM should be used for a more accurate assessment of organ dose.

A limitation of the study was that not all radiosensitive organs of the human body were investigated. Our aim was to examine radiosensitive organs that are primarily exposed in chest CT. These organs are considered to receive a higher dose in chest CT compared to partially irradiated organs or organs located beyond the boundaries of the planned image volume. Another limitation of this study was that organ doses were assessed on a CT scanner installed at our institution from single‐only vendor. Few studies have shown that normalized to CTDI_vol_ organ doses vary by less than 10% across different 64‐slice MDCT scanners for primarily irradiated organs.^34^, ^35^ It should be noted that the Discovery 750HD and Revolution HD scanner models from General Electric Healthcare, share the same operational specification characteristics including the dose index metric values provided by the two scanners.^36^

In conclusion, our results have shown that organ doses estimated with ImpactMC were in closer agreement to TLDs compared to NCICT. The advantage of ImpactMC is that organ dose assessment is performed on phantoms that resemble the realistic anatomy of the examined patients as opposed to NCICT methodology that incorporates an anatomical discrepancy between the phantoms used and patients. This study demonstrates that when dose calculations are performed for large‐scale patient cohorts in epidemiological studies, a more realistic representation of organ doses may be derived if patient‐specific dosimetry is employed.

## AUTHOR CONTRIBUTIONS

Antonios E. Papadakis collected and analyzed the data, interpreted the images, performed the statistical analysis, and drafted the initial manuscript. Vassiliki Giannakaki, John Stratakis, and Marios Myronakis collected the data. Habib Zaidi supported the study. John Damilakis conceived and supervised the study. All authors reviewed and approved the final manuscript.

## CONFLICT OF INTEREST STATEMENT

The authors have no conflict of interest to disclose.

## Supporting information

Supporting Information
