# Supplementary material for: Digital phantom versus patient‐specific radiation dosimetry in adult routine thorax CT examinations
Source: J Appl Clin Med Phys. 2024 May 22;25(7):e14389. doi: 10.1002/acm2.14389 (PMC11244670; doi:10.1002/acm2.14389)
Supplement: Supplementary file 1 — Supporting Information [file ACM2-25-e14389-s001.docx]

**Supplemental Material**

| **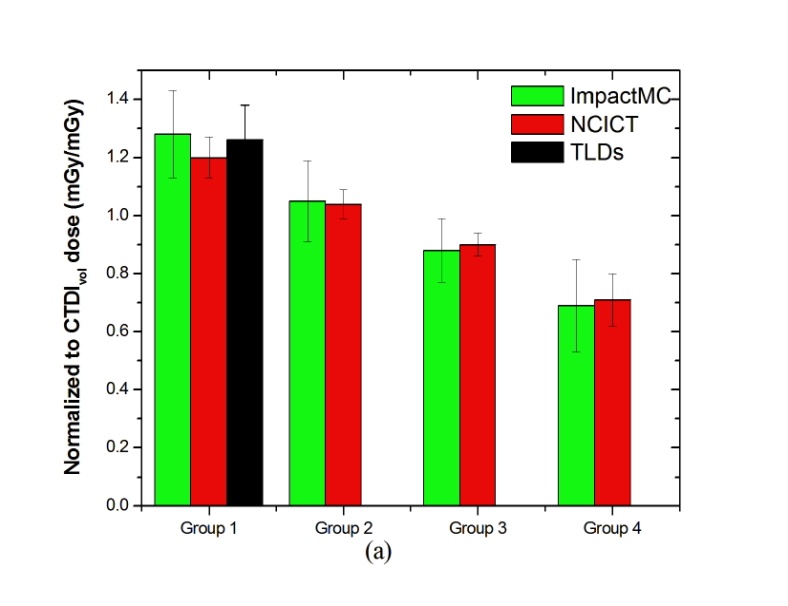** | **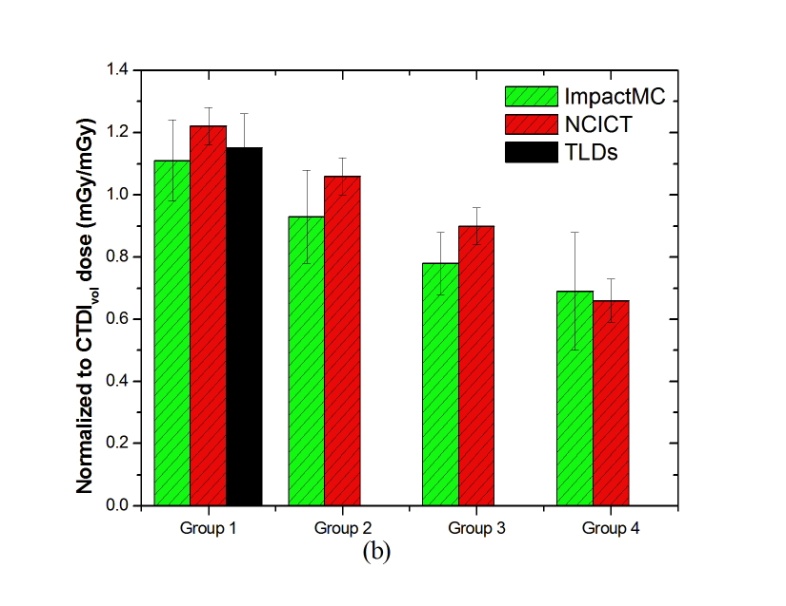** |
| --- | --- |
| **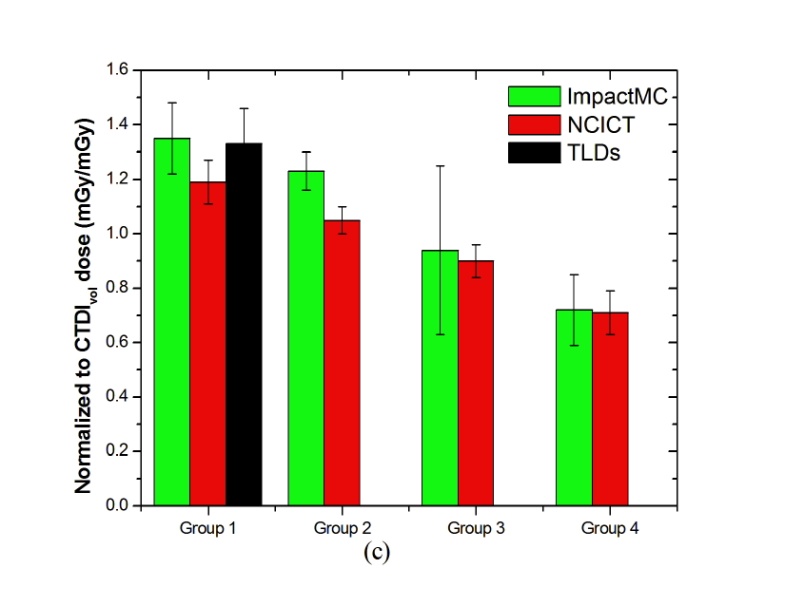** | **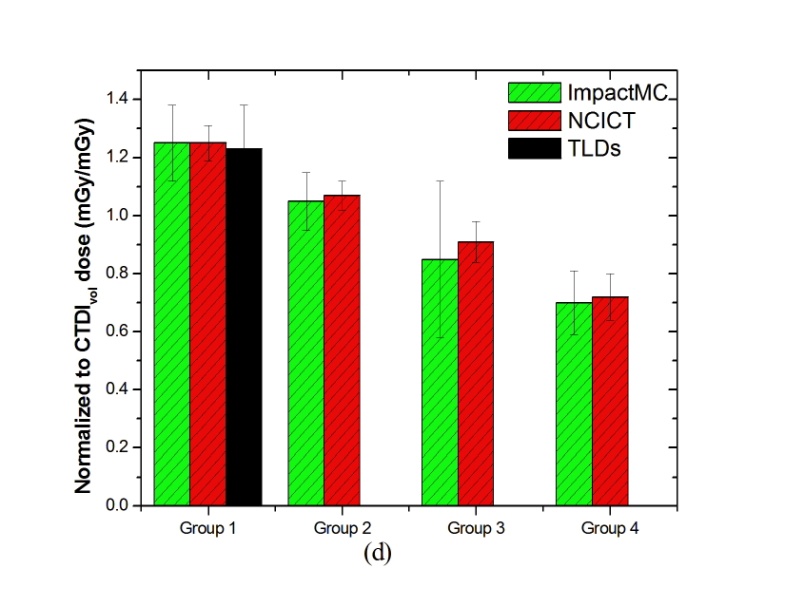** |

**Figure 1s.** Normalized to CTDI_vol_ esophagus dose assessed through different methodologies for each group in males with fixed mA (a) and TCM (b) and females with fixed mA (c) and TCM (d).

| **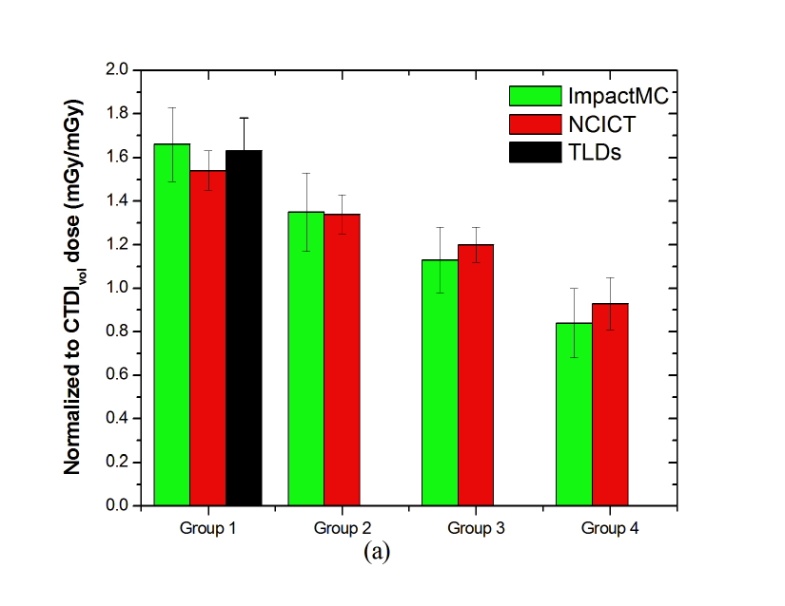** | **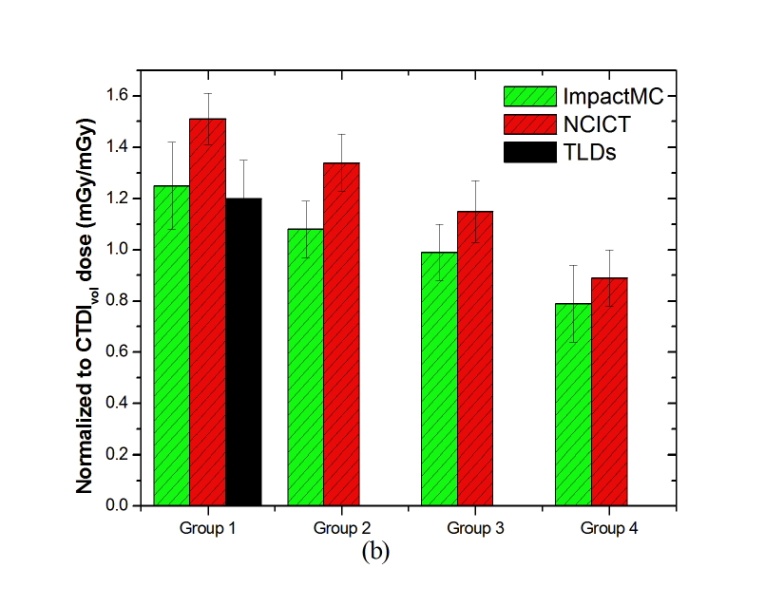** |
| --- | --- |
| **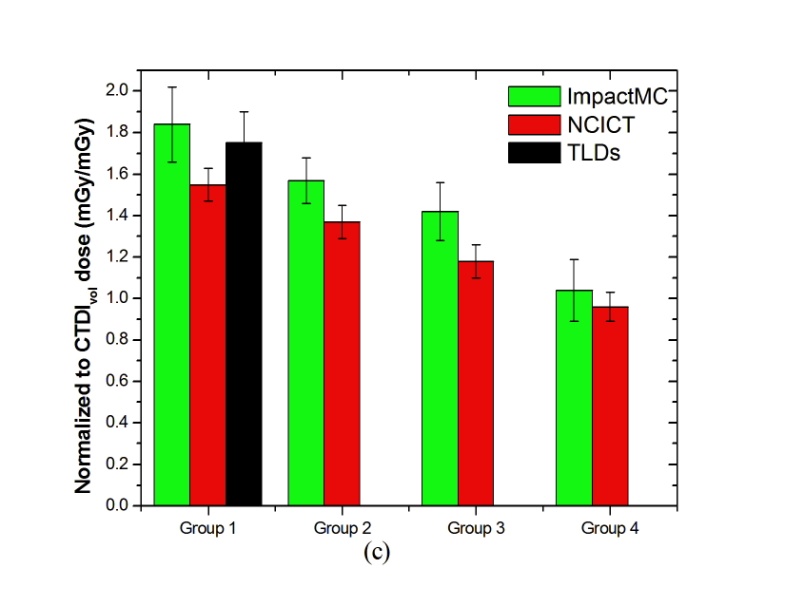** | **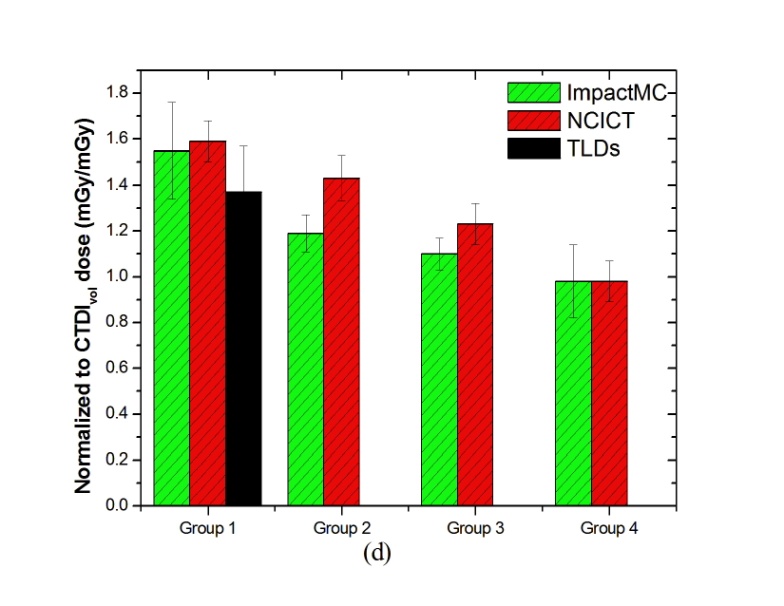** |

**Figure 2s.** Normalized to CTDI_vol_ heart dose assessed through different methodologies for each group in males with fixed mA (a) and TCM (b) and females with fixed mA (c) and TCM (d).

| **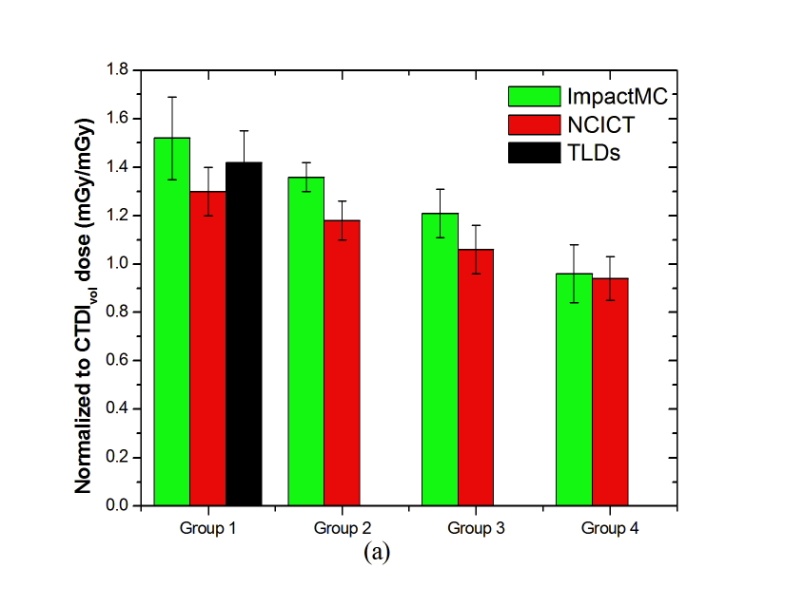** | **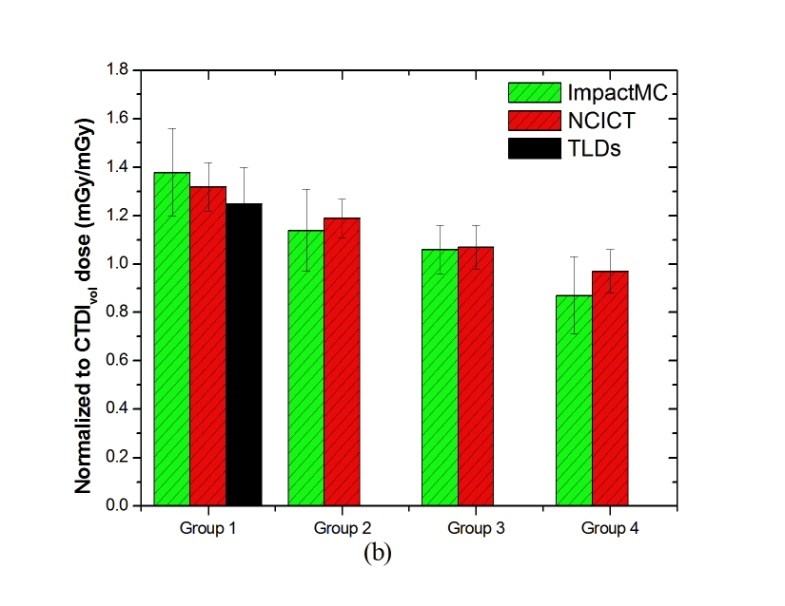** |
| --- | --- |

**Figure 3s.** Normalized to CTDI_vol_ breast dose assessed through different methodologies for each group in females with fixed mA (a) and TCM (b).

| **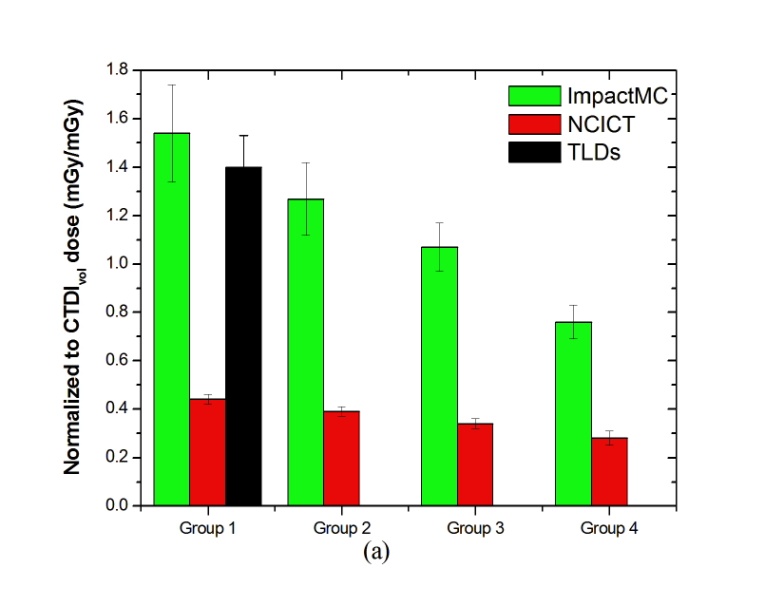** | **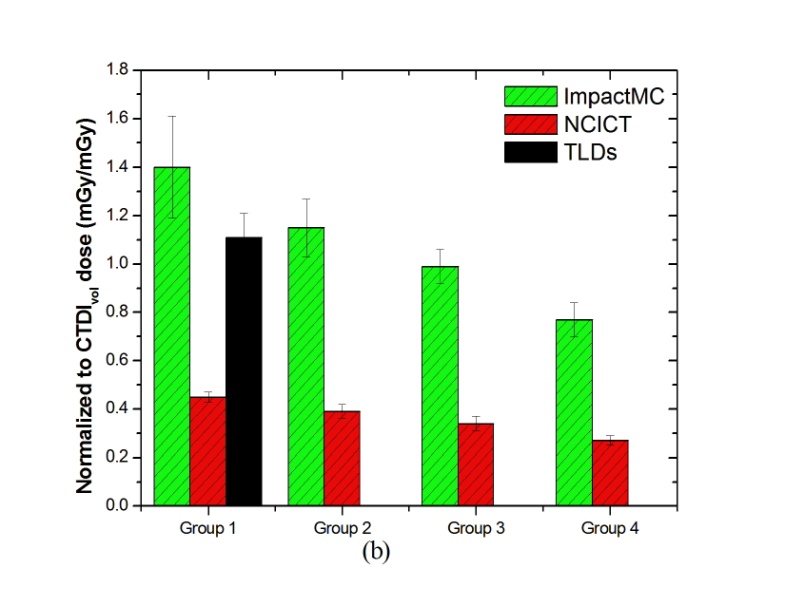** |
| --- | --- |
| **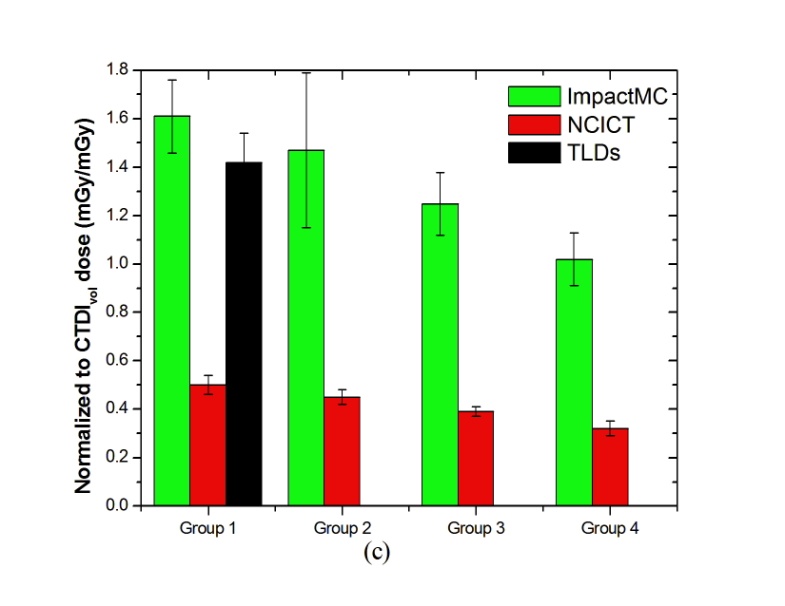** | **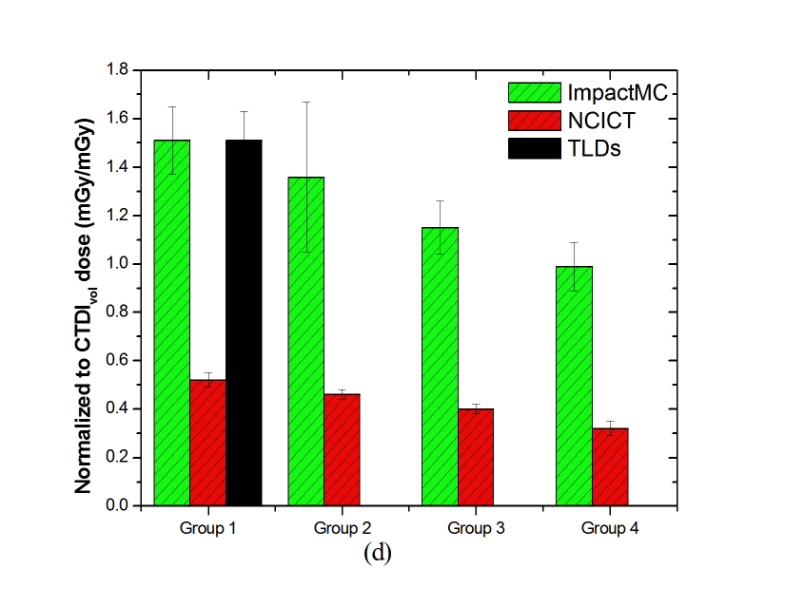** |

**Figure 4s.** Normalized to CTDI_vol_ active bone marrow dose assessed through different methodologies for each group in males with fixed mA (a) and TCM (b) and females with fixed mA (c) and TCM (d).

| 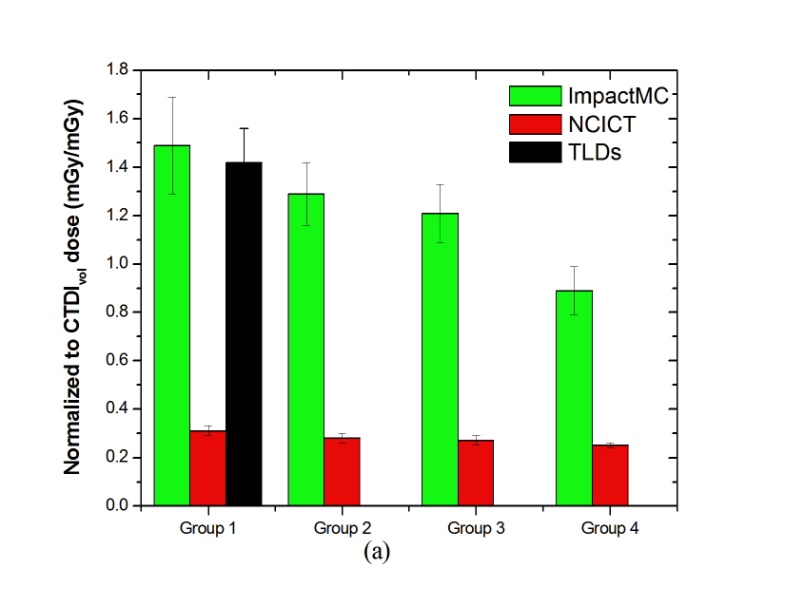 | 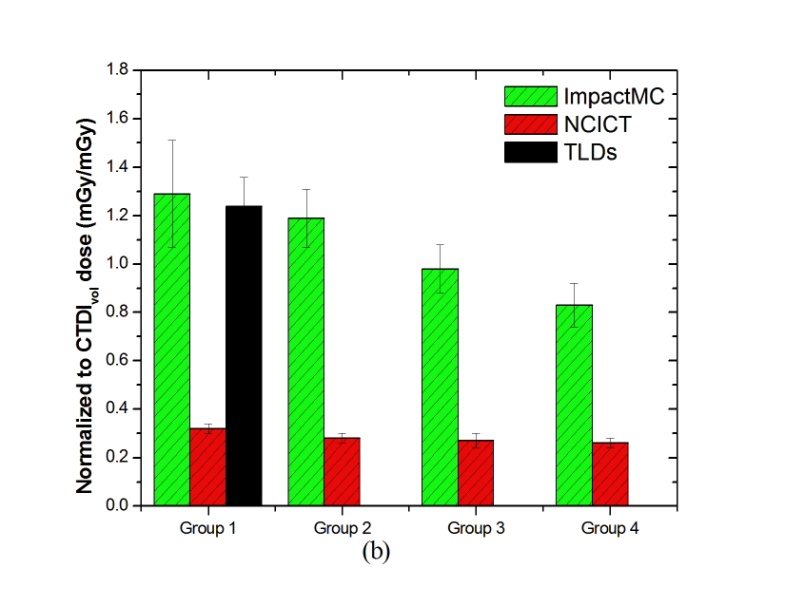 |
| --- | --- |
| 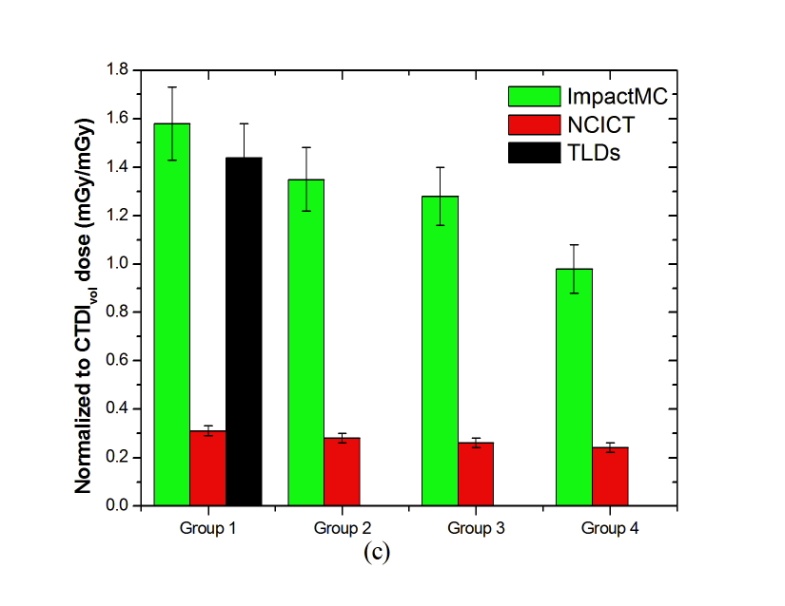 | 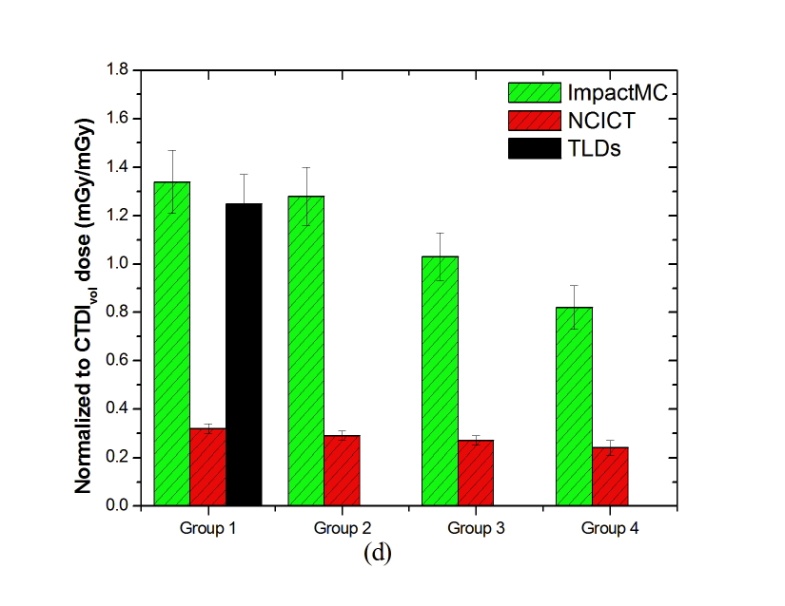 |

**Figure 5s.** Normalized to CTDI_vol_ skin dose assessed through different methodologies for each group in males with fixed mA (a) and TCM (b) and females with fixed mA (c) and TCM (d).
